# Gene expression modulation in TGF-β3-mediated rabbit bone marrow stem cells using electrospun scaffolds of various stiffness

**DOI:** 10.1111/jcmm.12533

**Published:** 2015-03-06

**Authors:** Qianping Guo, Chen Liu, Jun Li, Caihong Zhu, Huilin Yang, Bin Li

**Affiliations:** a Department of Orthopaedics, The First Affiliated Hospital of Soochow UniversitySuzhou, Jiangsu, China; b Orthopedic Institute, Soochow UniversitySuzhou, Jiangsu, China

**Keywords:** bone marrow stem cells, annulus fibrosus-derived stem cells, TGF-β3 treatment, gene expression, electrospun scaffolds, stiffness

## Abstract

Tissue engineering has recently evolved into a promising approach for annulus fibrosus (AF) regeneration. However, selection of an ideal cell source, which can be readily differentiated into AF cells of various regions, remains challenging because of the heterogeneity of AF tissue. In this study, we set out to explore the feasibility of using transforming growth factor-β3-mediated bone marrow stem cells (tBMSCs) for AF tissue engineering. Since the differentiation of stem cells significantly relies on the stiffness of substrate, we fabricated nanofibrous scaffolds from a series of biodegradable poly(ether carbonate urethane)-urea (PECUU) materials whose elastic modulus approximated that of native AF tissue. We cultured tBMSCs on PECUU scaffolds and compared their gene expression profile to AF-derived stem cells (AFSCs), the newly identified AF tissue-specific stem cells. As predicted, the expression of collagen-I in both tBMSCs and AFSCs increased with scaffold stiffness, whereas the expression of collagen-II and aggrecan genes showed an opposite trend. Interestingly, the expression of collagen-I, collagen-II and aggrecan genes in tBMSCs on PECUU scaffolds were consistently higher than those in AFSCs regardless of scaffold stiffness. In addition, the cell traction forces (CTFs) of both tBMSCs and AFSCs gradually decreased with scaffold stiffness, which is similar to the CTF change of cells from inner to outer regions of native AF tissue. Together, findings from this study indicate that tBMSCs had strong tendency to differentiate into various types of AF cells and presented gene expression profiles similar to AFSCs, thereby establishing a rationale for the use of tBMSCs in AF tissue engineering.

## Introduction

Intervertebral disc (IVD) degeneration is a major cause of low back pain, a common disease which affects about 80% of the population worldwide 1,2. Current treatments for degenerative disc disease (DDD), including discectomy and spinal fusion, only relieve the neurological symptoms, but do not prevent IVD degeneration. They may even cause degenerative post-discectomy spondylosis and adjacent vertebral degeneration 3,4. Recently, tissue engineering has received intensive attention as a novel approach for DDD treatment as it may reconstitute the functionality of native tissue. A number of techniques have been developed, with most of which focusing on the tissue engineering of nucleus pulposus (NP) 5. Such efforts, however, tend to fail without a well-functioned annulus fibrosus (AF), an essential IVD component for confining NP and maintaining physiological intradiscal pressure upon loading, to prevent disc re-herniation 5–7.Therefore, repairing/regenerating AF is a must for effective IVD repair/regeneration against DDD 8.

Cells play a central role in determining the quality of engineered tissues. Currently, AF cells or mesenchymal stem cells (MSCs) have been used in the majority of AF tissue engineering studies 9–15. However, mature AF cells quickly lose their phenotype and presented decreased collagen-II gene expression during *in vitro* expansion 16,17. Recently, AF-derived stem/progenitor cells (AFSCs) have been identified in humans, rabbits, rats and minipigs 18–21. Being AF tissue specific, AFSCs preferentially differentiate into various types of resident cells in native AF tissue and are therefore an ideal cell source for AF tissue engineering. However, it is difficult to harvest AFSCs through non-invasive approaches. It also remains unclear whether AFSCs isolated from degenerated IVDs are functionally capable or not. Isolation of AFSCs from healthy IVDs, on the other hand, is never advisable. In contrast, bone marrow MSCs (BMSCs) can be easily obtained in large quantity from bone marrow without traumatic operations, making them the most popular cell source for tissue engineering 22,23.

While MSCs of various origins all possess self-renewal and multi-potential differentiation capacity, they differ in many ways 24. Mesenchymal stem cells from adult tissues tend to be tissue specific, meaning that MSCs originated from a certain tissue preferentially and better differentiate into the type of cells residing in the particular tissue 24,25. Annulus fibrosus tissue composes of collagen-I, collagen-II and glycosaminoglycans such as aggrecan and their density varies by region 26–28. However, non-induced BMSCs expressed collagen-I, yet almost no collagen-II and aggrecan 29,30. Standard chondrogenic induction of BMSCs in monolayer culture resulted up-regulation of certain cartilage differentiation markers including COMP, PRELP, decorin and lumican, but not aggrecan and collagen-II 30. These imply that BMSCs per se may have inferior capability to differentiate into AF cells. Transforming growth factor β3 (TGF-β3) is effective in promoting cartilaginous matrix formation and has been widely used in cartilage and NP tissue engineering. Transforming growth factor-β3 treatment significantly increased the expression of cartilage-relevant genes, including collagen-II and aggrecan, in BMSCs 31. Indeed, it has been shown that in the presence of TGF-β3, BMSCs expressed a number of AF matrix-related genes resembling those in native AF tissue both in quality and quantity 32. Therefore, TGF-β3-mediated BMSCs (tBMSCs) hold potential as a candidate cell source for AF tissue engineering.

On the other hand, as a typical heterogeneous tissue the cellular phenotype, biochemical components and biomechanical characteristics of AF gradually change along the radial direction 26. The contents of collagen-I increases from inner to outer AF, while aggrecan and collagen-II levels decrease. Importantly, the distinctions in matrix composition of various AF regions are a result of the different types of cells, which produce different types of extracellular matrix (ECM) corresponding to the zone where they reside 26. The phenotype of AF cells gradually changes from more chondrocyte-like at inner region of AF to more fibroblast-like at outer region. It remains unclear whether tBMSCs, which are indeed pre-differentiated stem cells 31, could effectively differentiate into AF cells of various regions as AFSCs do.

In the past decade, numerous studies have shown that the mechanical property such as stiffness of cell culture substrate significantly affects the differentiation of stem cells and can direct their lineage specification 33–35. In this study, we have been suggested that tBMSCs respond similar to substrate stiffness as AFSCs do and may also be differentiated into AF-like cells using substrates whose stiffness is comparable to the various regions of native AF tissue. To this end, we prepared electrospun fibrous scaffolds from four biodegradable polyurethane materials (poly(ether carbonate urethane)-urea, PECUU), the elastic modulus of which approximates the stiffness of various AF regions 26,36. We then compared the differentiation efficiency between AFSCs and tBMSCs on these scaffolds at the gene level.

## Materials and methods

### Fabrication of electrospun PECUU scaffolds of different elastic moduli

The PECUU materials were synthesized according to a previous study 36.Their elastic modulus was measured using nanoindentation test according to our previously reported method 26. The PECUU solution (25 wt% in hexafluoroisopropanol) was loaded into a 2 ml syringe with an 18G needle and was fed at a constant rate of 0.5 ml/hr using a syringe pump (Longer Pump Co., Ltd., Baoding, Hebei, China). A positive voltage of 10 kV was applied to the needle using a high voltage power supply (Tianjin High Voltage Power Supply Co., Ltd., Tianjin, China). The distance between the collector and the needle tip was set at 15 cm. The scaffolds were dried under vacuum prior to use. The morphology of scaffolds was observed by scanning electron microscopy (SEM, S-4800; Hitachi, Kotyo, Japan). The SEM images of scaffolds were analysed using Image J software to determine the fibre diameters. The surface wettability of scaffolds was examined using a contact angle system (DSA25; KRÜSS, Hamburg, Germany). Five measurements were taken for each sample with a 4 μl drop of deionized water and the average of them was reported.

### Isolation of rabbit AFSCs and BMSCs

Rabbit AFSCs were isolated and cultured as previously described 18. AFSCs at passage 1 were used in this study. To isolate rabbit BMSCs, the femurs of New Zealand rabbits aged 3–6 months were taken out after anesthetized under a sterile environment. Then high glucose DMEM (SH30021.01B; Hyclone, Thermo Fisher Scientific, Hudson, NH, USA) containing 1000 U heparin, 100 U/ml penicillin and 100 μg/ml streptomycin was used to flush the bone marrow three times. The heparinized suspension was centrifuged at 55 g for 5 min. at room temperature. The bone marrow pellet was then re-suspended in 10 ml high glucose DMEM with 20% foetal bovine serum (FBS, SV30087.02; Hyclone), and filtered with a 200-mesh strainer. The cells were cultured at 37°C with 5% CO_2_. Non-adherent cells were removed by changing the media after 3 days, followed with changing the medium every other day 37. The third passage cells were used in this study. It should be noted that in all experiments, the two types of stem cells were from the same rabbit.

### Induced differentiation of AFSCs and BMSCs

Multi-differentiation potential of AFSCs and BMSCs were performed through induced differentiation for adipogenesis, osteogenesis and chondrogenesis. The cells were seeded at a density of 2 × 10^4^ cells/well in a 24-well plate in basic culture medium (DMEM with 10% FBS, 100 U/ml penicillin, 100 μg/ml streptomycin). They were subsequently cultured with adipogenic, osteogenic and chondrogenic induction medium when cells reached almost 80% confluence. For adipogenic induction, the cells were induced in an adipogenic induction medium consisting basic culture medium supplemented with 1 μM dexamethasome (D4902; Sigma-Aldrich), 10 μg/ml insulin (I6634; Sigma-Aldrich, St. Louis, MO, USA), 100 μM indomethacin (I7378; Sigma-Aldrich) and 0.5 mM isobutylmethylxanthine (IBMX) (I7018; Sigma-Aldrich). For osteogenic induction, the cells were induced in an osteogenic induction medium composed of basic culture medium supplemented with 0.1 μM dexamethasone, 0.2 mM ascorbic-2-phospate (A8960; Sigma-Aldrich) and 10 mM glycerol 2-phosphate (G8981; Sigma-Aldrich). For chondrogenic induction, a micro-mass method was used 38. The cells were cultured in a chondrogenic induction medium (RBXMX-90041; Cyagen Biosciences Inc., Santa Clara, CA, USA) which contained DMEM, 0.1 μM dexamethasone, 40 μg/ml l-proline, 100 μg/ml sodium pyruvate, 1% insulin, transferrin, sodium premix, 1% penicillin, streptomycin and fungizone and 10 ng/ml TGF-β3. After being cultured for 3 weeks (except 2 weeks for adipogenic induction), the cells were evaluated using Oil Red O staining for adipogenesis, Alizarin Red S staining for osteogenesis and Safranin O staining for chondrogenesis.

### Proliferation assays of AFSCs, BMSCs and tBMSCs on PECUU scaffolds

Poly(ether carbonate urethane)-urea substrates of different elastic modulus were cut into circles and placed into a 96-well plate. AFSCs and BMSCs were seeded at the density of 2000 cells/well. AFSCs were cultured on PECUU scaffolds in low glucose DMEM supplemented with 10% FBS. The BMSCs were divided into two groups. In one group (control group), BMSCs were cultured in low glucose DMEM supplemented with 10% FBS. In another group (induced group), BMSCs were cultured in a chondrogenic differentiation medium (Catalog No. RBXMX-90041; Cyagen, DMEM, 0.1 μM dexamethasone, 40 μg/ml l-proline, 100 μg/ml sodium pyruvate, 1% insulin, transferrin, sodium premix and 1% penicillin, streptomycin and fungizone, 10 ng/ml TGF-β3) 31. At 1, 3, 5 and 7 days after cell seeding, the samples were incubated with 20 μl MTS reagent in 200 μl PBS for 2 hrs. The absorbance at 490 nm was measured using a microplate reader (BioTek, Winooski, VT, USA).

### Morphology observation of AFSCs, BMSCs and tBMSCs on PECUU scaffolds

After 1, 3 and 7 days of culture on PECUU scaffolds, the morphology of cells was evaluated using cytoskeleton staining and SEM. For cytoskeleton staining, cells on scaffolds were rinsed with PBS twice, fixed in 4% paraformaldehyde and permeabilized with 0.1% Triton X-100 for 5 min., rinsed with PBS twice, followed by staining with FITC-phalloidin (Enzo Biochem, New York, NY, USA) and DAPI (Roche, Basel, Switzerland) for visualizing F-actin and nuclei respectively and observation under a fluorescence microscope (Zeiss Axiovert 200; Carl Zeiss Inc., Thornwood, NY, USA). For SEM observation, the samples were rinsed with PBS, fixed with 2.5% glutaraldehyde for 2 hrs and then rinsed with deionized water three times. The samples were then dehydrated through graded ethanol from 50% to 100% for 10 min. each, dried and sputter-coated with gold. Then they were observed using SEM at an accelerating voltage of 3 kV.

### Gene expression analysis of AFSCs, BMSCs and tBMSCs on PECUU scaffolds

The PECUU scaffolds were cut to fit into the wells of a 24-well plate and sterilized by Co-60 irradiation. The cells were seeded at a density of 5 × 10^4^ cells/well. AFSCs were cultured in basic medium, while tBMSCs were cultured in TGF-β3 mediated medium. In a control group, BMSCs were cultured in basic medium. After 2 weeks, total RNA were extracted using TRIZOL isolation system (Invitrogen, Thermo Fisher Scientific, Hudson, NH, USA) following the manufacturer’s protocol. cDNA was synthesized using a Revert-Aid™ First-Strand cDNA Synthesis Kit (K1622; Fermentas, Thermo Fisher Scientific, Hudson, NH, USA) and oligo (dT) primers for 60 min. at 42°C on a RT-PCR system (Eastwin Life Science, Beijing, China). Real-time quantitative PCR (RT-qPCR) was performed with a Bio-Rad CFX96™ Real-Time System using the SsoFast™ EvaGreen Supermix Kit (Bio-Rad Laboratories, Berkeley, CA, USA). The relative gene expression level was analysed using the △△C_T_ method by referring to the gene expression of AFSCs on PECUU-1 scaffolds after being normalized to the gene expression of Glyceraldehyde-3-phosphate dehydrogenase (GAPDH) as the internal control. The forward and reverse primer sequences for collagen-I, collagen-II, aggrecan and GAPDH were designed using the mRNA sequences deposited in NCBI GenBank (Table1).

**Table 1:** Sequences of primers for RT-PCR

Gene	Sequence	Accession number
Collagen-I	Forward: 5′-CTGACTGGAAGAGCGGAGAGTAC-3′	AY633663
	Reverse: 5′-CCATGTCGCAGAAGACCTTGA-3′	
Collagen-II	Forward: 5′-AGCCACCCTCGGACTCT-3′	NM_001195671
	Reverse: 5′-TTTCCTGCCTCTGCCTG-3′	
Aggrecan	Forward: 5′-ATGGCTTCCACCAGTGCG-3′	XM_002723376
	Reverse: 5′-CGGATGCCGTAGGTTCTCA-3′	
GAPDH	Forward: 5′-ACTTTGTGAAGCTCATTTCCTGGTA-3′	NM_001082253
	Reverse: 5′-GTGGTTTGAGGGCTCTTACTCCTT-3′	

### Cell traction force microscopy analysis

After being cultured on PECUU scaffolds for 2 weeks, AFSCs and tBMSCs were removed by 0.25% trypsin and plated on fluorescent beads-embedded, collagen-coated polyacrylamide gels at the density of 3000 cells/dish. The cells were allowed to attach and spread on the gel for 6 hrs before cell images were taken for cell traction force microscopy (CTFM) measurement according to a published protocol 26. Pictures of individual cells were taken as a phase contrast image and a fluorescence image, namely, ‘force-loaded’ image. A fluorescence image of the fluorescent beads as ‘null-force’ in the same view was taken after cells were removed. The CTFs were then computed using a customer-written MATLAB program based on the three images 39.

### Statistical analysis

All data are represented as mean ± SD. Statistical analyses were performed with SPSS software (IBM, Armonk, NY, USA). Non-parametric anova was used to analyse the contact angle, gene expression level and CTFs results. An unpaired Student’s *t*-test was also used where appropriate. Difference between two groups is considered statistically significant if *P* < 0.05.

## Results

### Fabrication and characterizations of electrospun PECUU scaffolds

**Figure 1:**
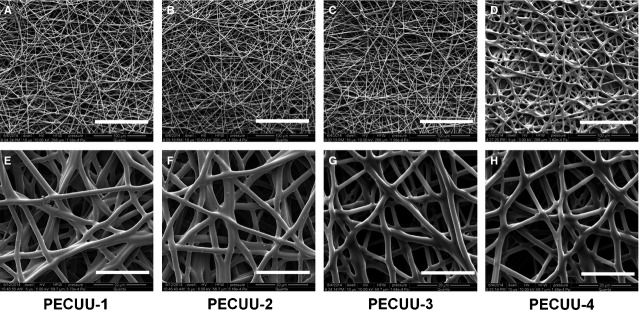
Scanning electron microscopy (SEM) pictures of the electrospun poly(ether carbonate urethane)-urea (PECUU) nanofibrous scaffolds of different elastic modulus. Scale bars, (A–D) 100 μm; (E–H) 20 μm.

Four kinds of PECUU materials were used in this study. The elastic modulus of them, as measured using nanoindentation test, was 13.4 ± 1.7, 6.4 ± 0.5, 5.1 ± 0.2 and 2.5 ± 0.2 MPa for PECUU-1, PECUU-2, PECUU-3 and PECUU-4 respectively (Fig. S1). Four sets of PECUU scaffolds with randomly oriented micro-fibres, which might mimic the fibrous nature of native AF matrix, were then fabricated using electrospinning technique (Fig.1). As estimated using SEM imaging, the average diameter of PECUU scaffolds was 2.5–2.7 μm (Fig. S2).The contact angles of all scaffolds only slightly differed, ranging from 101.6 to 107.3°, indicating similar surface wettability of them (Fig. S3).

### Isolation and multi-potential differentiation of AFSCs and BMSCs

**Figure 2:**
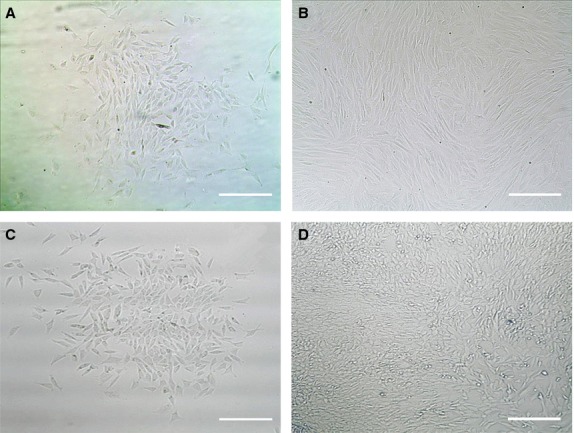
Phase contrast images of primary and the third passage bone marrow stem cells (BMSCs; A and B) and primary and the first passage AFSCs (C and D). Scale bars, 20 μm.

**Figure 3:**
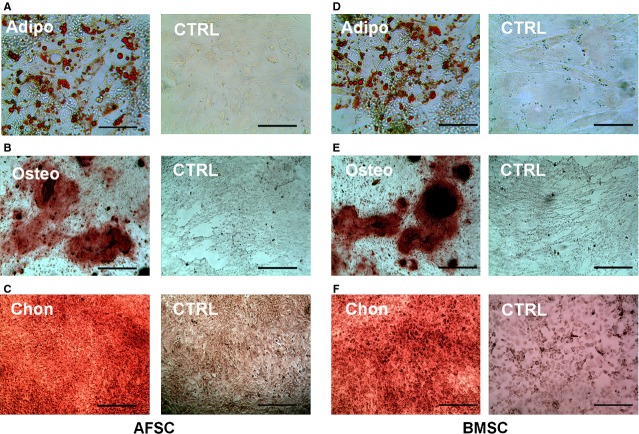
Multiple differentiation potential tests of AFSCs (A–C) and bone marrow stem cells (BMSCs; D–F). (A and D) Adipogenic differentiation at 2 weeks for AFSCs and BMSCs respectively. Lipid droplets stained using Oil Red were seen in the induced cells. (B and E) Osteogenic differentiation at 3 weeks for AFSCs and BMSCs respectively. Calcified deposits stained by the Alizarin Red S staining were seen in the induced cells. (C and F) Chondrogenic differentiation at 3 weeks for AFSCs and BMSCs respectively. Sulphated proteoglycans stained using Safranin O was mostly seen in the induced cells. Scale bars, (A and D) 50 μm; (B, C, E, F) 200 μm.

When the primary rabbit AFSCs and BMSCs were cultured in the growth medium with 20% FBS, they started to form colonies after 3 days. The cells were harvested and sub-cultured when they reached about 80% confluence. Both AFSCs and BMSCs were spindle-shaped and fibroblast-like, typical morphology of MSCs (Fig.2). The multi-differentiation potential of AFSCs and BMSCs were examined by culturing them in the induction medium of adipogenesis, chondrogenesis and osteogenesis. Liquid globules, as stained using Oil Red O, were observed surrounding the cells after 2 weeks of culture in adipogenic induction medium. Calcium deposits were identified by Alizarin Red S staining after 3 weeks of culture in osteogenic induction medium. Meanwhile, large amount of sulfated proteoglycans was detected by staining with Safranin O after 3 weeks of culture in chondrogenic induction medium (Fig.3).

### Growth of AFSCs and tBMSCs on PECUU scaffolds

**Figure 4:**
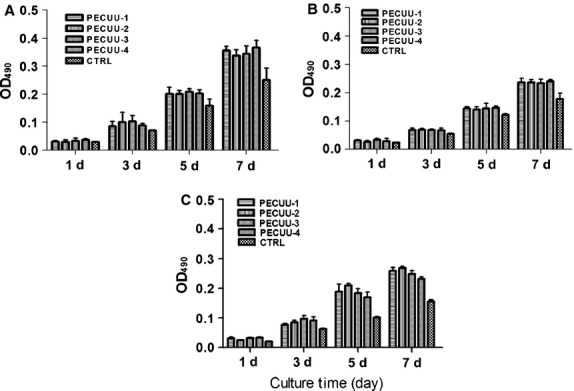
Proliferation of AFSCs (A), tBMSCs (B) and non-treated BMSCs (C) on poly(ether carbonate urethane)-urea (PECUU) scaffolds of different elastic modulus after 1, 3, 5 and 7 days of culture.

**Figure 5:**
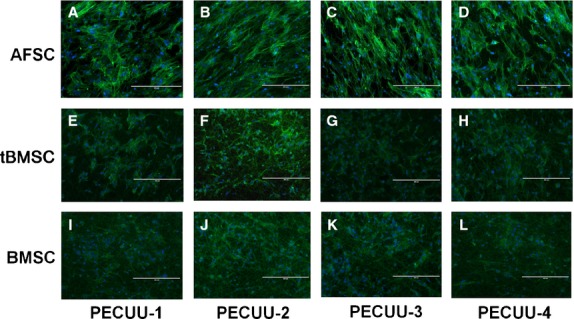
Fluorescence microscopy images of AFSCs (A–D), tBMSCs (E–H) and BMSCs (I–L) subjected to FITC-phalloidin (green) and DAPI (blue) staining after 3 days of culture on poly(ether carbonate urethane)-urea (PECUU) scaffolds of different elastic modulus. Scale bars, 400 μm.

**Figure 6:**
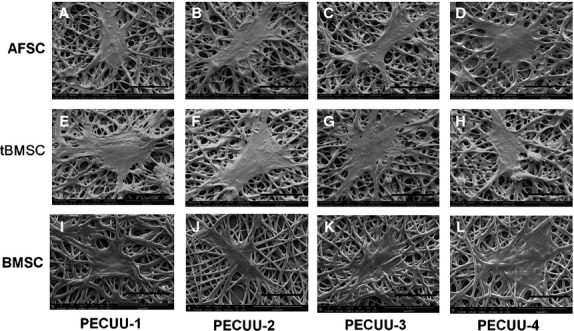
Scanning electron microscopy (SEM) pictures of AFSCs (A–D), tBMSCs (E–H) and BMSCs (I–L) after 3 days of culture on poly(ether carbonate urethane)-urea (PECUU) scaffolds of different elastic modulus. Scale bars, 50 μm.

Cell growth on PECUU scaffolds was measured using MTS assay at 1, 3, 5 and 7 days of culture. Clearly, AFSCs, tBMSCs and untreated BMSCs (as control) grew well and continued proliferating on all PECUU scaffolds regardless of their elastic modulus. Interestingly, all cells grew slightly faster on PECUU scaffolds compared to the regular tissue culture plates which served as a control, likely because of the nano-/micro-fibrous and porous microstructure of the electrospun scaffolds 40 (Fig.4). In addition, it appears that AFSCs grew faster than tBMSCs and BMSCs on PECUU scaffolds irrespective of their stiffness. The morphology of cells, shown by F-actin staining, also clearly indicates that all the cells attached and grew well on PECUU scaffolds (Fig.5). Closer observation of the cell morphology using SEM discovered that the cells attached well on the scaffolds and naturally stretched along the nano-/micro-fibres (Fig.6).

### Gene expression analysis

**Figure 7:**
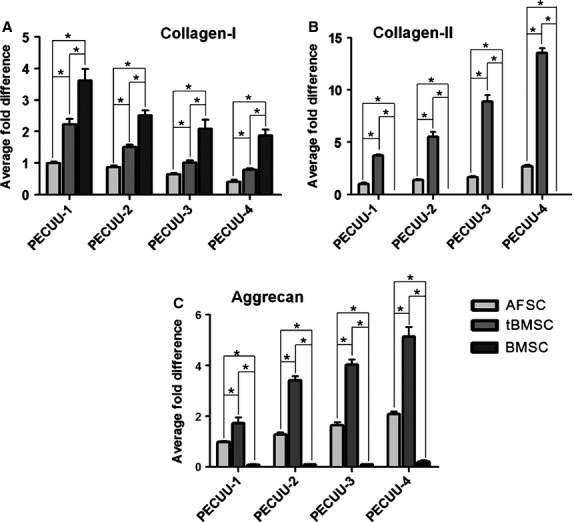
The expression of AF-related genes collagen-I (A), collagen-II (B) and aggrecan (C) in AFSCs, tBMSCs and BMSCs cultured on poly(ether carbonate urethane)-urea (PECUU) scaffolds of different elastic modulus for 2 weeks. Note that in (B), the expression of collagen-II in BMSCs was too little to be displayed. Asterisk (*) indicates significant difference between groups (*P* < 0.05).

After 2 weeks of culture on PECUU scaffolds, the gene expression levels of collagen-I, collagen-II and aggrecan in the cells were determined using RT-qPCR. Clearly, the expression of collagen-I in AFSCs and tBMSCs increased with the stiffness of PECUU substrate (Fig.7A). On the other hand, the expression of collagen-II and aggrecan genes showed exactly an opposite trend (Fig.7B and C). For example, the expression of collagen-I in AFSCs on PECUU-1 (Young’s modulus = 13.4 MPa) was 2.5 times higher than that on PECUU-4 (2.5 MPa). However, the expression of collagen-II and aggrecan on PECUU-4 was 2.5 times and 2.0 times, respectively, higher than those on PECUU-1. Similarly, the expression of collagen-I in tBMSCs on PECUU-1 was 2.5 times higher than on PECUU-4, whereas the expression of collagen-II and aggrecan on PECUU-4 was 3.5 times and 3.0 times, respectively, higher than on PECUU-1. As for BMSCs growing on PECUU scaffolds without the mediation of TGF-β3, the expression of collagen-I of them on PECUU-1 was 2 times higher than on PECUU-4 (Fig.7A). However, there was very few or even no detectable expression of other AF tissue relevant genes, including collagen-II (Fig.7B) and aggrecan (Fig.7C), in BMSCs on PECUU scaffolds of different stiffness. In addition, it is clear that the expression levels of collagen-I, collagen-II and aggrecan genes in tBMSCs were all significantly higher than those in AFSCs regardless of the stiffness of PECUU scaffolds.

### CTFM analysis

**Figure 8:**
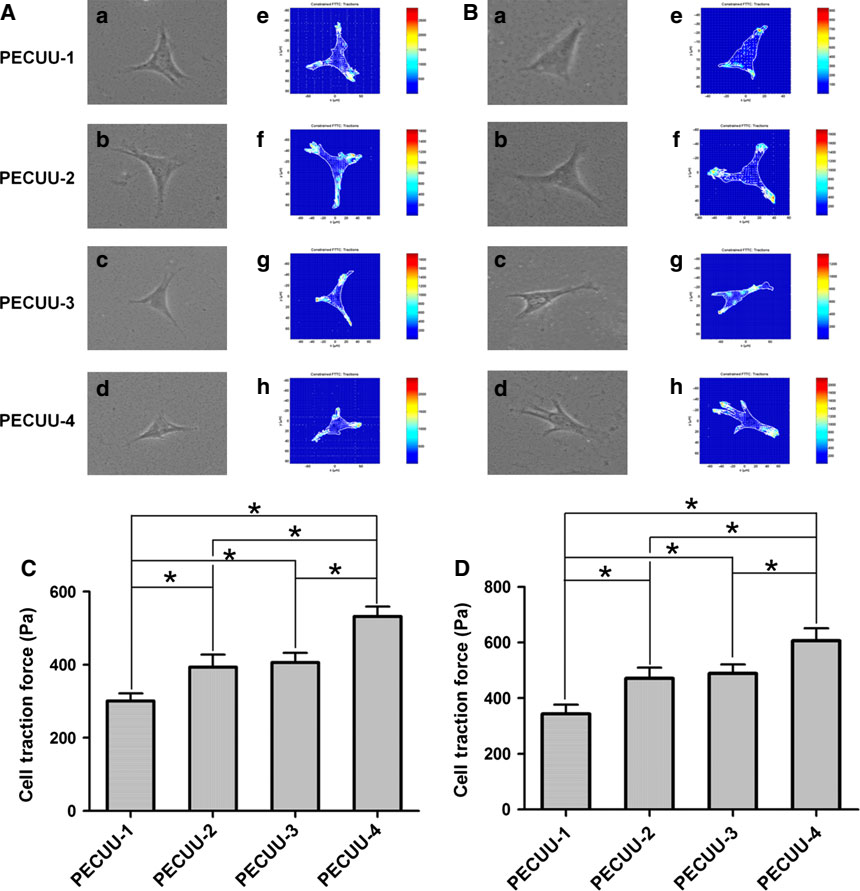
Cell traction force microscopy (CTFM) measurement of AFSCs and tBMSCs cultured on poly(ether carbonate urethane)-urea (PECUU) scaffolds of different elastic modulus for 2 weeks. (A and B) CTFM for measuring CTFs of AFSCs and tBMSCs respectively. (a–d) Phase contrast images of cells; (e–h) CTF maps of corresponding cells. (C and D) The computed CTFs of AFSCs and tBMSCs respectively. All data are presented as mean ± SEM. Asterisk (*) indicates significant difference between groups (*P* < 0.05, *n* ≥ 20).

The CTFs of AFSCs and tBMSCs on PECUU scaffolds of different Young’s modulus were measured using CTFM technology (Fig.8A and B). Apparently, the CTFs of AFSCs gradually increased with the decrease of PECUU substrate stiffness, being 303.3 ± 97.9, 394.0 ± 159.0, 406.2 ± 129.6 and 532.1 ± 128.7 Pa when they were cultured on PECUU-1, PECUU-2, PECUU-3 and PECUU-4 scaffolds respectively (Fig.8C). Similarly, the CTFs of tBMSCs were 343.8 ± 148.7, 471.3 ± 171.6, 489.2 ± 145.7 and 606.7 ± 197.7 Pa on PECUU-1, PECUU-2, PECUU-3 and PECUU-4 scaffolds respectively (Fig.8D).

## Discussion

To date, major challenge still remains towards preparation of engineered AF alternatives that are comparable to native AF tissue both biologically and functionally, mainly because of the tremendous complexity of AF at cellular, biochemical, microstructural and biomechanical levels 26,41. Being AF tissue specific, the newly identified AFSCs could be a valuable source for AF tissue engineering. However, applications of AFSCs tend to suffer from the limited availability of AF tissue. In contrast, BMSCs, which can be easily extracted from bone marrow in large quantity, hold promise for AF tissue engineering applications. Nevertheless, BMSCs have poor ability to differentiate into AF-like cells in terms of the expression of AF relevant genes such as collagen-II and aggrecan 29.

Reportedly, application of TGF-β3 as a chondrogenesis inducer strongly promoted the expression of chondrogenic genes such as collagen-II and aggrecan in BMSCs, while down-regulated the level of osteogenic marker collagen-I expression 42–44. In this study, the differentiation potential of TGF-β3-mediated BMSCs, *i.e*. tBMSCs, towards AF-like cells was studied by comparing with AFSCs. Since AF is generally considered as a fibrocartilage tissue mainly consisting of collagen-I, collagen-II and proteoglycans and cells with the characteristics of fibrochondrocytes, the expression of collagen-I, collagen-II and aggrecan was used to evaluate the efficacy of AF-related differentiation of stem cells. Excitingly, with the mediation of TGF-β3, the expression of collagen-II and aggrecan genes was remarkably improved in BMSCs cultured on PECUU scaffolds, although the expression of collagen-I was slightly lowered. In addition, the expression of collagen-I, collagen-II and aggrecan genes was markedly higher in tBMSCs compared with those in AFSCs. These findings are in line with a previous report by Steck *et al*., in which BMSCs under TGF-β3 mediation in 3D culture showed a similar gene expression profile as native IVD tissue and histological appearance close to fibrocartilage 32.

It is now well known that the mechanical property such as stiffness of substrate strongly affects the behaviours such as adhesion, proliferation, differentiation and migration of cells 33,35,45. For instance, human BMSCs were effectively differentiated into bone, muscle or neuronal lineages when they were cultured on stiff, medium or soft substrates, the stiffness of which was close to that of corresponding native tissues 33. We have previously shown that the elastic modulus of rabbit AF tissue gradually increases from the inner, middle to outer regions 26. Correspondingly, the inner AF mainly consists of collagen-II and proteoglycans, whereas the outer AF mainly contains collagen-I. Therefore, in this study we fabricated PECUU substrates of different elastic modulus to mimic the stiffness gradient of AF tissue. We found that on low modulus PECUU scaffolds, the expression of collagen-I gene in both AFSCs and tBMSCs was relatively low, whereas the expression of collagen-II and aggrecan genes was relatively high. These findings echo the results of a few previous studies 46,47. In other words, on the soft PECUU scaffolds, both AFSCs and tBMSCs tended to differentiate into the cells which resembled the inner AF cells. However, on the stiff PECUU scaffolds, they preferentially differentiated into the cells whose characteristics were similar to the outer AF cells. Such substrate stiffness-dependent modulation of gene expression was also seen in the differentiation of TGF-β mediated stem cells towards smooth muscle cells 35. Based on these findings, we speculate that the BMSCs might be firstly pre-differentiated into IVD-like precursor cells upon TGF-β3 mediation 32, and further differentiated into various AF-like cells according to the stiffness of PECUU substrate which resembled the different regions of native AF tissue.

In addition to gene expression characterizations, we also used another approach, *i.e*. CTF measurement through CTFM, to evaluate the differentiation of stem cells. CTFs are the mechanical forces that a cell generates against the underlying substrate 48. Since different populations of cells can be clearly distinguished in the CTF distribution profile, CTFM may serve as an effective biophysical approach for characterizing cell differentiation by determining the CTF changes of cells 49. Previously, we found that the CTF of AF cells gradually changed along the radial direction of AF 26. This provides a novel way to distinguish inner, middle and outer AF cells regions using CTFM in addition to examining their morphology and gene expression. Clearly, the changes of CTF of both AFSCs and tBMSCs as a result of the stiffness difference of PECUU scaffolds are similar to the region-dependent CTF distribution profile of AF cells as shown in our previous study 26. Such differentiation-associated mechanical changes have also been seen in other types of stem cells 50,51. Therefore, the findings from CTF measurement also imply that both AFSCs and tBMSCs underwent differentiation — as a function of the stiffness of PECUU scaffold — towards AF-like cells in various regions of native AF tissue.

In summary, we have found that in response to the stiffness variation of PECUU scaffolds, tBMSCs, *i.e*. the pre-differentiated BMSCs under TGF-β3 mediation, showed expression profiles of ECM genes (collagen-I, collagen-II and aggrecan) similar to the AF cells at different regions of native AF tissue. In addition, the differentiation efficiency of tBMSCs into AF-like cells, represented by the level of ECM gene expression, appeared to be similar to if no better than AFSCs, the endogenous stem cells in native AF tissue. Being the first to compare the differentiation efficiency of tBMSCs and AFSCs, this study provides a rationale for the use of tBMSCs, along with scaffolds of varying stiffness, for AF tissue engineering. Certainly, there are still several limitations in this study. First, we did not obtain PECUUs with elastic modulus in the kPa range because of the nature of PECUU chemistry; therefore, the stiffness of PECUU scaffolds did not closely mimic that of native AF tissue. Second, the AFSCs and tBMSCs were cultured in a static environment in this study. It is unclear how such cells respond to substrate stiffness in a physiologically relevant, loaded environment. Finally, in the electrospun scaffolds used in this study the micro-fibres were disorganized and did not resemble the aligned fibrous structure in native AF tissue. As a matter of fact, in an ongoing study we have found that on aligned fibres the expression of collagen-I and aggrecan genes in AFSCs was increased, yet the expression of collagen-II gene was almost not affected (Fig. S4), which was in line with the result from a previous study 52. Our following studies will aim to further improve the differentiation efficacy of tBMSCs towards AF-like cells by culturing them on aligned fibrous scaffolds whose stiffness closely matches native AF tissue under an environment with dynamic mechanical loading which resembles the physiological situation of AF *in vivo*. In addition to the anabolic genes, a number of catabolic genes/proteins as well as inflammatory factors will also be checked for better understanding the cellular responses against substrate stiffness changes.

## Supporting Information

**Figure S1** The Young’s modulus of PECUU materials measured using nanoindentation test. Asterisk (*) indicates significant difference between groups (*P* < 0.05, *n* ≥ 15).

**Figure S2** The fibre diameters of PECUU scaffolds. No significant difference is seen between various scaffolds.

**Figure S3** Water contact angle (WCA) measurement of electrospun PECUU scaffolds of different elastic modulus. (A) The sessile water drop images on the scaffolds. (B) WCAs of the scaffolds.

**Figure S4** The expression of Col-I, Col-II and Aggrecan genes in AFSCs cultured on aligned and random PECUU-3 scaffolds.
