# Supplementary figures and images for: Gene expression modulation in TGF-β3-mediated rabbit bone marrow stem cells using electrospun scaffolds of various stiffness
Source: J Cell Mol Med. 2015 Mar 6;19(7):1582–92. doi: 10.1111/jcmm.12533 (PMC4511356; doi:10.1111/jcmm.12533)

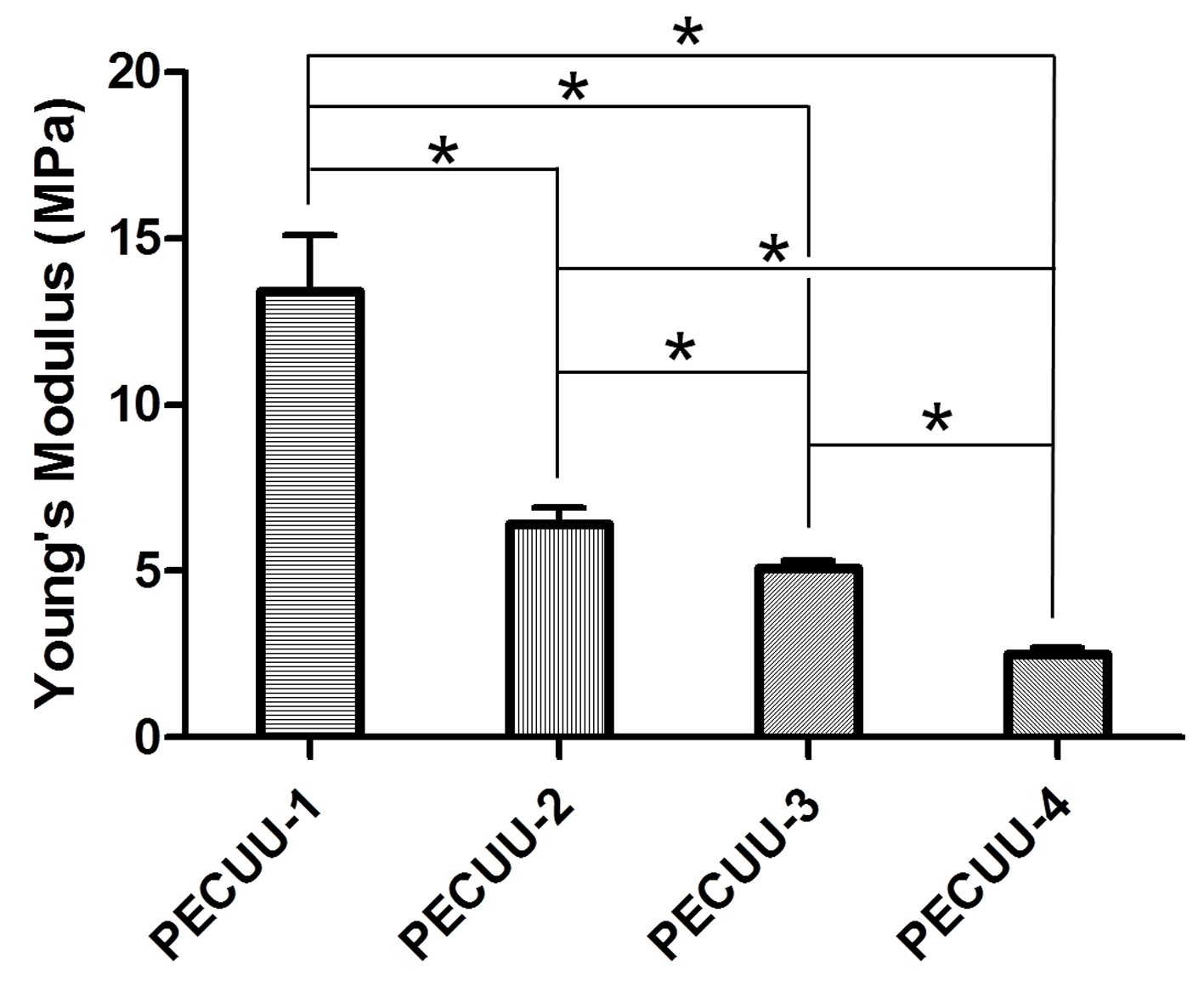

Supplement: Supplementary file 1 [file jcmm0019-1582-sd1.tif]

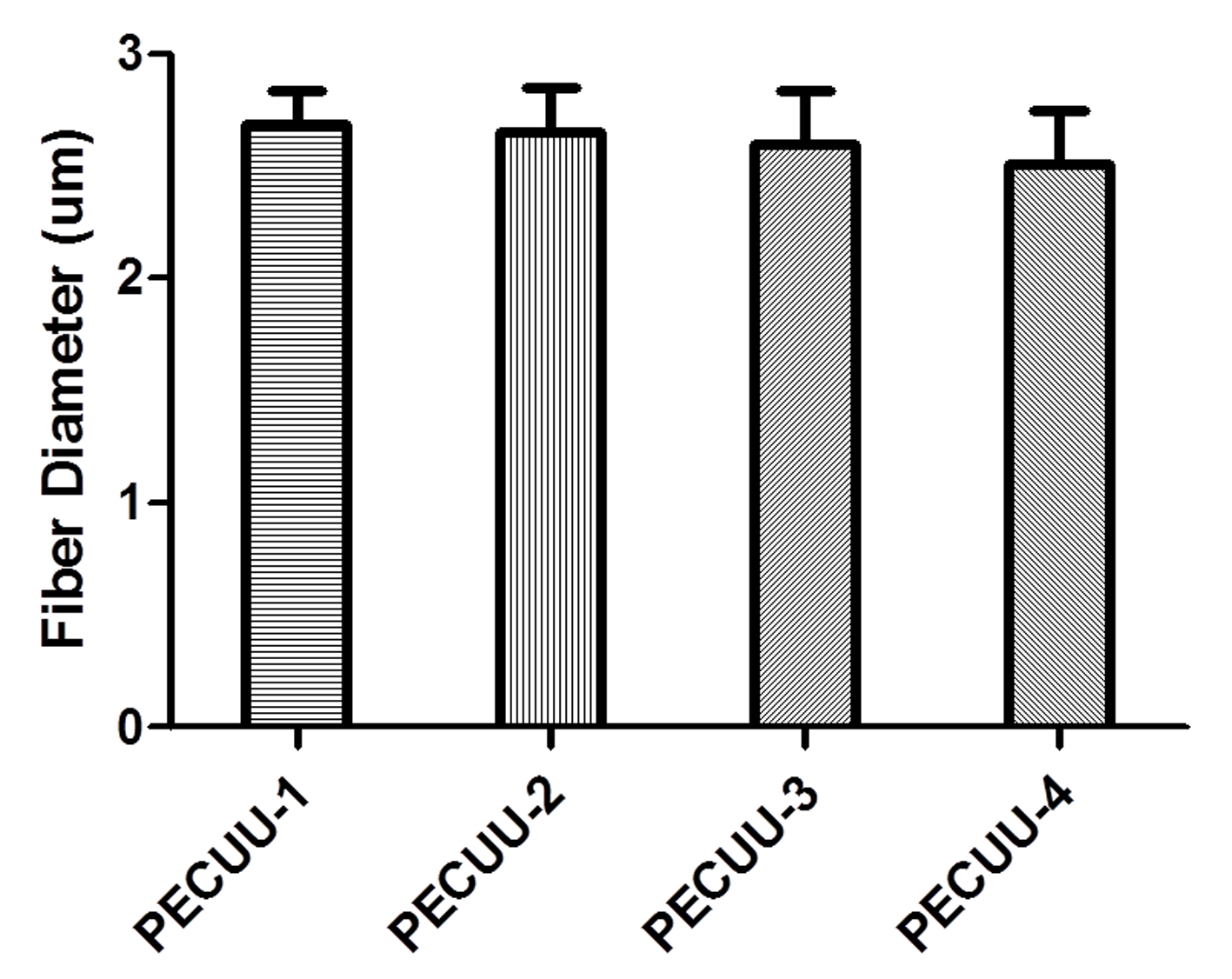

Supplement: Supplementary file 2 [file jcmm0019-1582-sd2.tif]

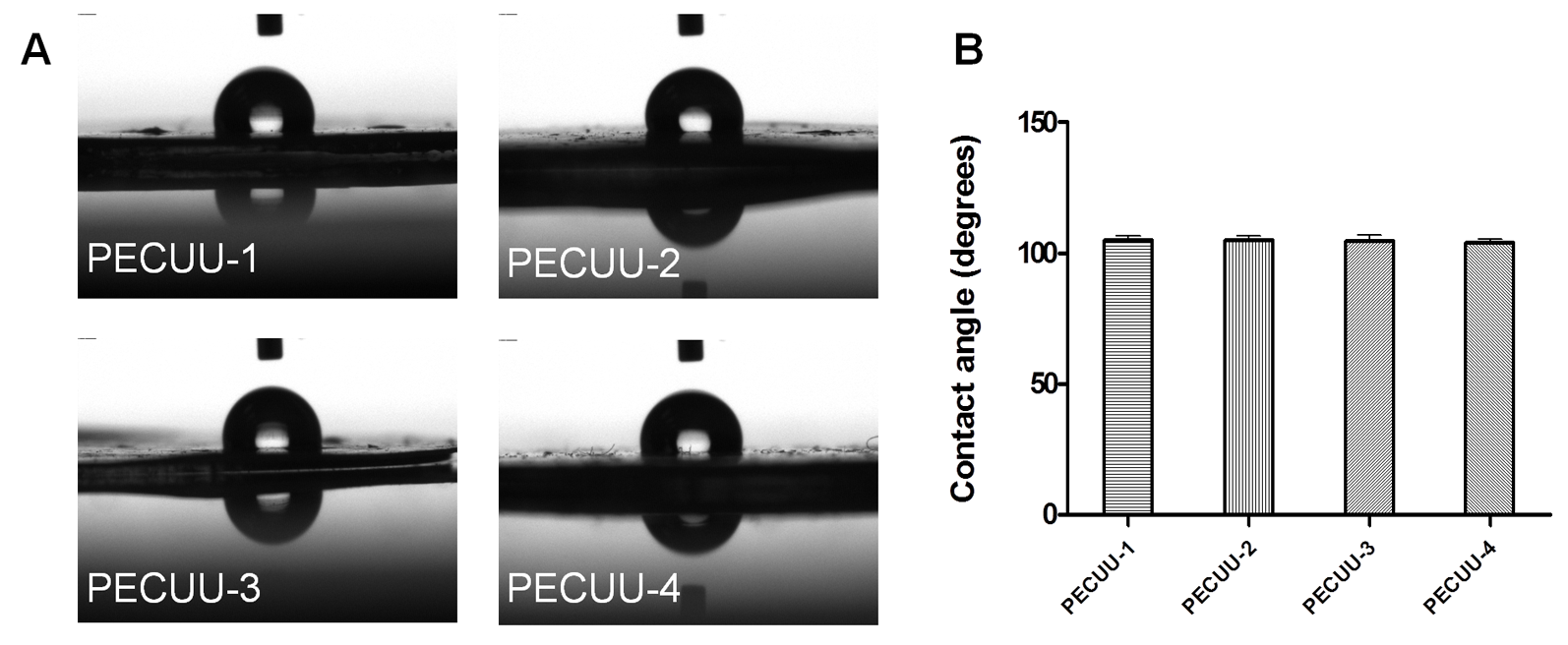

Supplement: Supplementary file 3 [file jcmm0019-1582-sd3.tif]

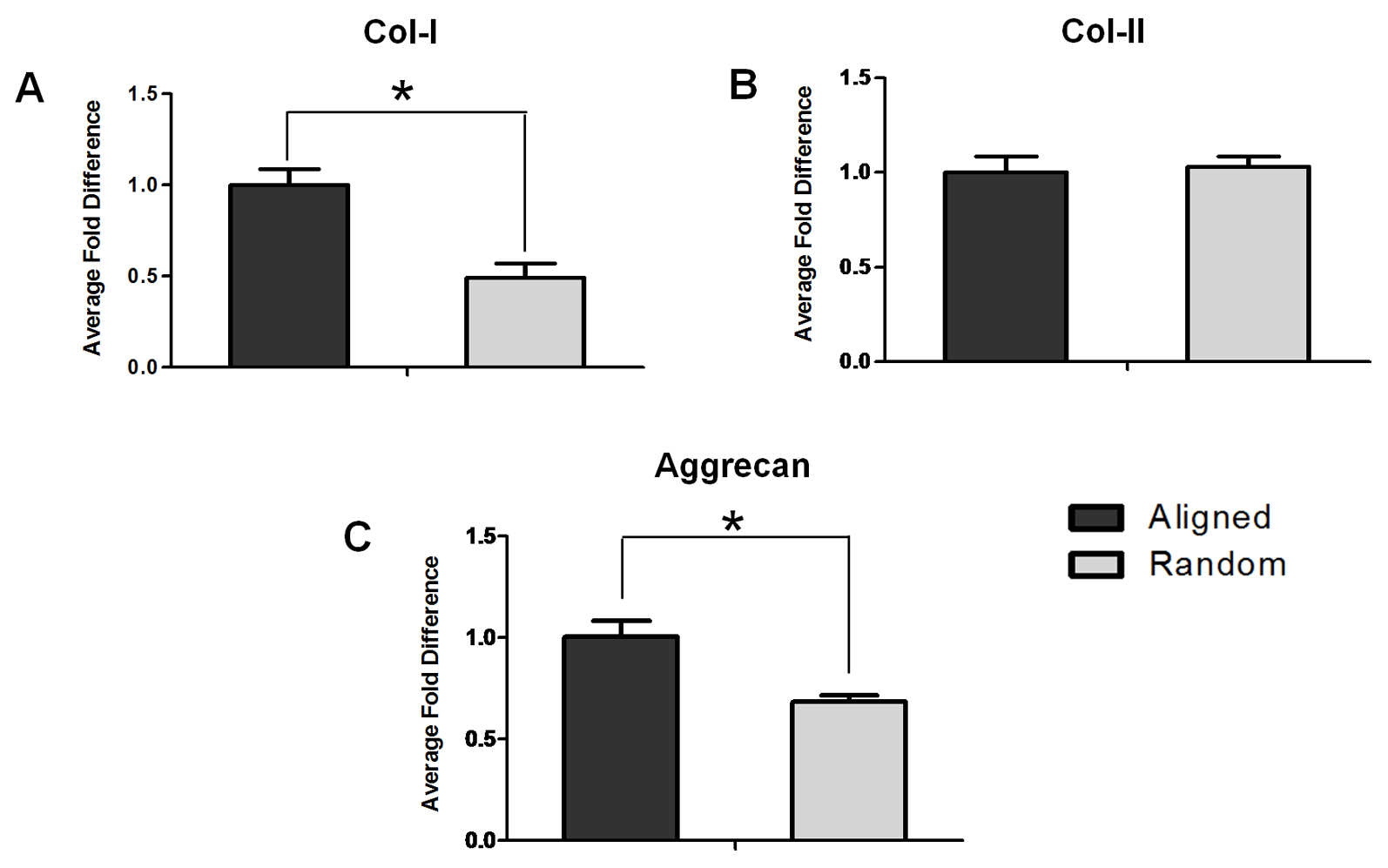

Supplement: Supplementary file 4 [file jcmm0019-1582-sd4.tif]
